# Transcriptomic analysis reveals overdominance playing a critical role in nicotine heterosis in *Nicotiana tabacum* L.

**DOI:** 10.1186/s12870-018-1257-x

**Published:** 2018-03-22

**Authors:** Maozhu Tian, Qiong Nie, Zhenhua Li, Jie Zhang, Yiling Liu, Yao Long, Zhiwei Wang, Guoqing Wang, Renxiang Liu

**Affiliations:** 1 Key Laboratory of Tobacco Quality in Guizhou province, Guizhou University, Guiyang, 550025 China; 2 College of Tobacco, Guizhou University, Guiyang, 550025 China; 3 National Maize Improvement Center of China, Beijing Key Laboratory of Crop Genetic Improvement, China Agricultural University, Beijing, 100193 China

**Keywords:** Nicotine, Heterosis, *Nicotiana tabacum* L., RNA-seq

## Abstract

**Background:**

As a unique biological phenomenon, heterosis has been concerned with the superior performance of the heterosis than either parents. Despite several F1 hybrids, containing supernal nicotine content, had been discovered and applied to heterosis utilization in *Nicotiana tabacum* L., nevertheless, the potential molecular mechanism revealing nicotine heterosis has not been illustrated clearly.

**Result:**

Phenotypically, the F1 hybrids (Vall6 × Basma) show prominent heterosis in nicotine content by 3 years of field experiments. Transcriptome analysis revealed that genes participating in nicotine anabolism (*ADC, PMT, MPO, QPT, AO*, *QS*, *QPT, A622, BBLs*) and nicotine transport (*JAT2, MATE1 and 2, NUP1 and 2*) showed an upregulated expression in the hybrid, a majority of which demonstrated an overdominant performance. RT-PCR confirmed that nicotine anabolism was induced in the hybrid.

**Conclusions:**

These findings strongly suggest that nicotine synthesis and transport efficiency improved in hybrid and overdominance at gene-expression level played a critical role in heterosis of nicotine metabolism.

**Electronic supplementary material:**

The online version of this article (10.1186/s12870-018-1257-x) contains supplementary material, which is available to authorized users.

## Background

Heterosis refers to a unique biological phenomenon that hybrid is superior to either parents in growth potential, yield, resistance, and fitness [1, 2]. Previously, three quantitative genetic hypotheses explained heterosis: the dominance [1], overdominance [2], and epistasis [3]. Since then, heterosis is a major concern for both biologists and breeders. Despite the successful agronomic exploitation of heterosis in several crops, especially hybrid rice and hybrid maize, the molecular mechanisms underlying crop heterosis are yet to be elucidated [4].

Recently, omics technology has been comprehensively applied in studying the molecular mechanism of heterosis, genome-wide changes in gene expression [5–9], siRNA [10], DNA methylation [11], and histone modifications [8, 9] for hybrids and their parents have been already compared. The genome-wide comparative transcriptional profiling has been exploited to study heterosis in *Arabidopsis* [12], rice [13], maize [14], soybean [15], *Brassica napus* [16], grape [17], petunias [18], *Medicago sativa* [19], and eucalyptus [20]. Using genome-wide association studies (GWAS) approach, several superior alleles that contributed to yield heterosis were identified in rice [21], significant locus and candidate genes that contributed to flowering period and biomass-related heterosis were verified in *Arabidopsis* [22, 23]. In addition, the mapping for heterosis quantitative trait loci on yield in rice hybrids has been reported [4, 24]. However, none of studies have investigated heterosis in *Nicotiana tabacum* L., which is an important model species in Solanaceae.

Nicotine, a main alkaloid in *Nicotiana tabacum* L., played a pivotal role in *Solanaceae* plant defense against diseases and pests. Accompanied by biosynthesis in roots [25], nicotine is translocated into leaf by xylem transport [26]. Nicotine is synthesized by two different nitrogen-containing rings, the pyrrole ring and the pyridine ring, which are derived from different synthetic pathways [27]. For pyrrole ring, nicotine biosynthesis could initiate directly through the decarboxylation of ornithine by ODC to form putrescine [28] or indirectly through ADC-mediated decarboxylation of arginine to form putrescine [29]. Then, putrescine is catalyzed by PMT, a key regulatory enzyme for nicotine anabolism, to form N-methylputrescine [30]. Finally, N-methylputrescine is catalyzed by MPO, forming the direct precursor of nicotine. For pyridine ring, QPT is the rate-limiting enzyme in nicotine anabolism [31]. Recently, enzymes encoded by genes *A622* and *BBLs*, were deduced to function in the last steps of nicotine synthesis [32–34].

*Nicotiana tabacum* L. is a classic allotetraploid crop species developed by natural hybridization of two progenitor species, *Nicotiana sylvestris* and *Nicotiana tomentosiformis* [35], interspecific and intraspecific of *nicotina* genus all had sufficient amount of heterosis [36–39]. Heterosis has been widely studied with respect to growth rate, flower days, plant height, and leaf yield [40–42]. Reportedly, some hybrids in flue-cured varieties, between *N. rustica* and *N. tabacum*, showed a positive heterosis in the nicotine content [43, 44]. However, molecular mechanisms of nicotine heterosis have not been illustrated in tobacco. Hence, this study aimed to reveal it by transcriptome analysis using model plant *Nicotiana tabacum* L. To our knowledge, this is the first study to describe nicotine heterosis in plants on a transcriptomic scale.

## Methods

### Plant materials and nicotine analysis

Tobacco hybrid F1, derived by crossing the varieties of Va116 (female parent, *flue-cured tobacco*) and Basma (male parent, Oriental tobacco). Va116 and Basma were collected from Tobacco Institute at Chinese Academy of Agriculture Sciences (CAAS). The seeds were sowed in floating plates and grown in a greenhouse until the seedlings contains five euphylla. Using a randomized block design, all seedlings were planted with three replicates, with planting distance and space of 110 × 55 cm on the experimental base at Guizhou University in 2013, 2014, and 2015, and it must be stated that they were topped 70 days after transplanting. The leaves and roots were collected at 7-day intervals from 56 to 91 days after transplantation. All leaves and roots samples were collected as follows: at least 10 of them were picked from the mother plants, respectively, mixed as a biological repetition, and three repetitions were used for the experiment. Leaf nicotine were obtained from the extract of dry samples and measured by gas chromatography according to described by Shoji T. et al. [45]. In addition, root samples were collected in 2015 for genes expression analysis (7-day intervals from 63 to 91 days after transplantation) and transcriptomics (samples collected 77 days after transplantation).

### RNA isolation and sequencing

The root tissues were treated with Total RNA purification Kit (LC Science, TRK-1001) to extract the total RNA, the whole process was done at the manufacturer’s suggested protocols. All the total RNA samples were controlled in a high quality condition, in which A260/A280 > 2, RIN value > 7. Subsequently, the mRNA was purified from 5 μg total RNA by poly-T oligo-attached magnetic beads, to obtain fragments of ~ 100–400 bp. The RNA fragments were used for the first strand-cDNA synthesis by using reverse transcriptase and random primers. Followed, second-strand cDNA was synthesized by using DNA polymerase I and RNase H. Agilent 2100 Bioanalyzer and ABI StepOne Plus Real-Time PCR System were used for the qualitative and quantitative analyses of all libraries. Three cDNA libraries, with 200 bp insert size, were selected for sequencing with the Illumina HiSeq 2500 platform (Illumina Inc., San Diego, CA, USA).

### Transcriptomics data processing and analysis

The raw reads were preprocessed. In this process, the adaptor sequences, short sequences with a length < 25 nt, low quality sequences were removed. After preprocessing, the obtained reads were mapped to the *Nicotiana tabacum* sequenced cultivar K326 genome using the splice-aware mapping tool, Tophat2 [46]. The intermediate result files generated by Tophat2 were used as the input data sets of Cufflinks [47]. Subsequently, the libraries, that were assembled by Cufflinks and Cuffmerge [47], were used to merge these assemblies in order to find the novel expressed gene loci. Then, the abundance in expression (FPKM) of all genes was estimated by Cufflinks. The novel expressed genes with longest transcript ≥200 bp were annotated using the NCBI NR database <ftp://ftp.ncbi.nih.gov/blast/db> and KEGG pathway (www.genome.jp). The differential expression genes (DEG) were analyzed using the statistical tool R and DESeq package [48]. The *P*-values of DEGs were corrected by Benjamini–Hochberg FDR (false discovery rate) correction [49]. The over-representation of GO terms and KEGG pathways of DEGs were identified using the Goatools (https://github.com/tanghaibao/Goatools) and R GO package [50] respectively. The *P*-value of the enriched analyses was corrected by the FDR method.

### Real-time PCR

To verify the expression levels of tobacco genes obtained from RNA sequencing, Real-time PCR (RT-qPCR) experiments were conducted to quantify the expression of a few random genes. Quantitative Real-time PCR was performed using SYBR Premix Ex Taq Kit (Takara) according to the manufacturer’s protocol. The RT-qPCR reactions were conducted on Applied Biosystems 7500 Real-Time PCR System (Life Technologies Corporation, Beverly, MA, USA). Two Actin genes were used as the endogenous reference genes, and the expression level of each gene was normalized against the Actin-2 gene. Genes and primers for the qPCR are listed in Additional file 1: Table S1. To calculate the relative expression level of individual gene, 2^−ΔΔ Ct^ method [51] was adopted. The expression data of all genes, which had being generated by the real-time PCR experiments, were displayed as average values with standard error appended.

### Statistical analysis

Field experiment design was randomized with three replicates (see “Plant materials and nicotine analysis” part). The nicotine content was used for ANOVA, which was performed using Duncan’s test in SPSS software Ver.16.0. (*P* < 0.05). Over high-parent heterosis (OPH), mid-parent heterosis (MPH), and below low-parent heterosis (BPH) was calculated as follows: $\mathrm{OPH}\ \left(\%\right)=\left(\frac{\mathrm{F}1-\mathrm{HP}}{\mathrm{HP}}\right)\times 100$, $\mathrm{MPH}\ \left(\%\right)=\left(\frac{\mathrm{F}1-\mathrm{MP}}{\mathrm{MP}}\right)\times 100$, $\mathrm{BPH}\ \left(\%\right)=\left(\frac{\mathrm{F}1-\mathrm{LP}}{\mathrm{LP}}\right)\times 100$, where; F1 = performance of the hybrid, HP = high-value parent, MP = mid-parent average$\left[\frac{\left(\mathrm{parent}1+\mathrm{parent}2\right)}{2}\right],$ LP = low-value parent.

## Results

### Nicotine content significantly increased in roots of F1 hybrids

**Fig. 1:**
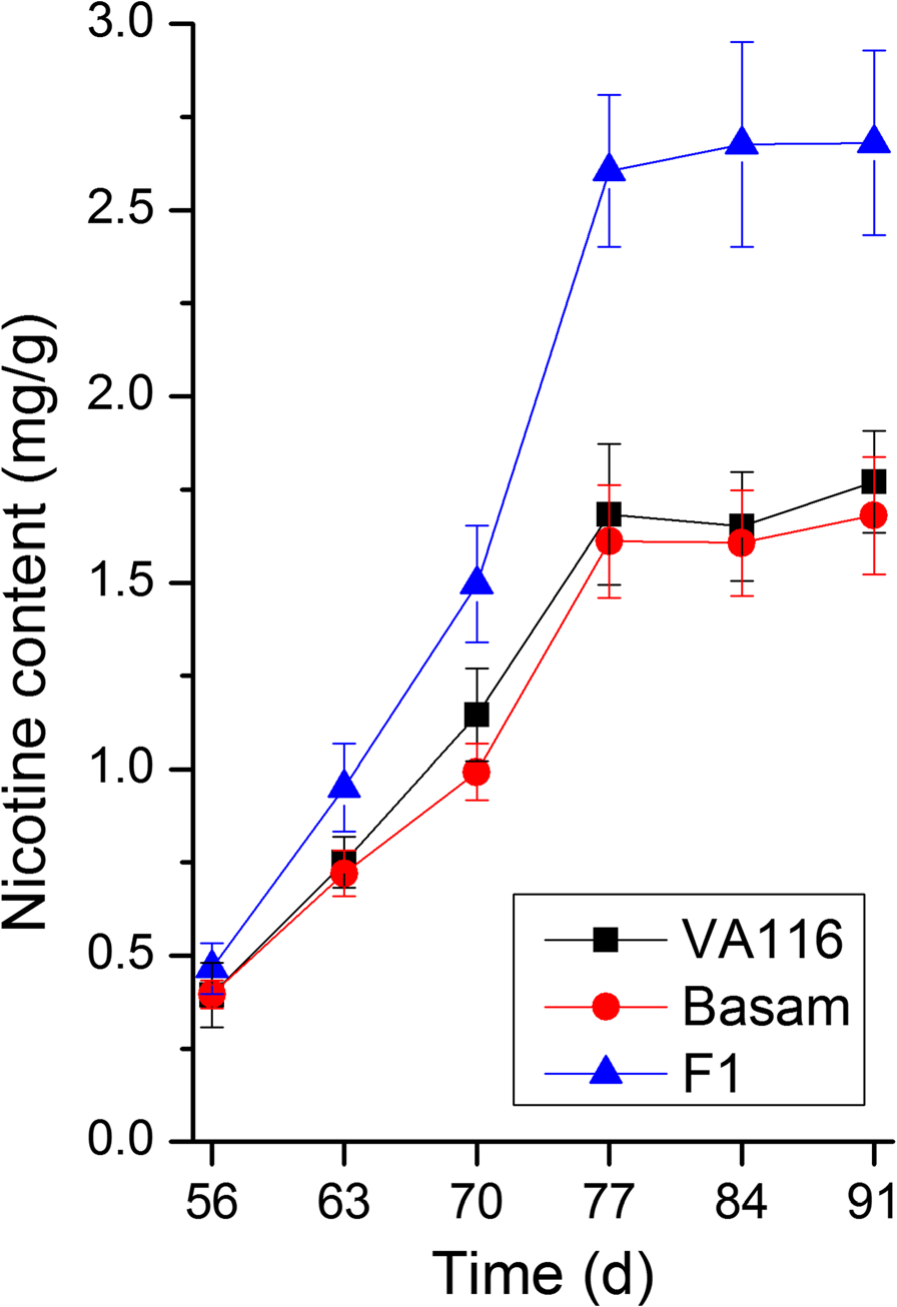
Nicotine content of F1 hybrids and their parents during leaf development

**Fig. 2:**
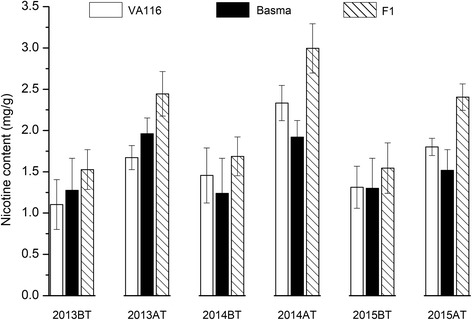
Heterosis of the F1 hybrid in *Nicotiana tabacum* L. of the two developmental stages, BT and AT, which represent before topping and after topping, respectively

The nicotine content of parental and its hybrid was measured from 56 to 91 days after transplant, and the results were summarized in Fig. 1. During development, a rapid increase in the nicotine content was observed during the initial 3 weeks (56–77 days, especially 70–77 days) with both parents and its hybrid, while a gradual increase was observed subsequently (77–91 days). The growth rate of nicotine content for the hybrid exceeded that of the parents. As shown in Fig. 2, continuous 3 years of the experimental study revealed that the nicotine content of the hybrids was significantly higher than that of parents after topping. However, no significant difference was observed between them before topping. Table 1 displayed less difference in OPH, MPH, and BPH heterosis values before topping; conversely, these were increased significantly after topping. The mid-parent (MPH) value (MPV) was ~ 40% higher.

**Table 1:** Heterosis of nicotine content of hybrid combination(Va116 × Basma)in tobacco

Years	Developmental stage	Mid-parent heterosis	Over high-parent heterosis	Below low-parent heterosis
2013	BT	11.48	3.92	20.24
	AT	34.56	24.65	46.18
2014	BT	10.39	2.13	20.11
	AT	40.84	28.38	55.99
2015	BT	2.83	2.36	3.31
	AT	44.82	33.48	58.26

### Transcriptome differences of leaves between parents and F1 hybrids

RNA sequencing technique was employed to study the nicotine heterosis of tobacco. Three paired-end libraries, VA116, Basma, and their hybrid, were constructed and sequenced on the Illumina HiSeq 2000 platform. The quality assessment of the sequencing data was shown in Additional file 2: Table S2. Three libraries are listed in Table 2. Clean reads accounted for > 95% of the total reads with error rates of 0.02%, Q20 > 96%, Q30 > 91%. The read counts and genomic mapping parameters of the three libraries are listed in Additional file 1: Table S1, which showed that 80.59–82.64% clean reads were mapped on the *N. tabacum* L. K326 reference genome [52].

**Table 2:** Comparison results of clean sequences on reference genomes

Sample	Clean reads	Total mapped	Multiple mapped	Uniquely mapped	Reads map to +	Reads map to -	Non-splice reads	Splice reads
VA116	69282386	56937342 (82.18%)	1149403 (1.66%)	55787939 (80.52%)	27915758 (40.29%)	27872181 (40.23%)	39876131 (57.56%)	15911808 (22.97%)
Basma	76348022	63090417 (82.64%)	1516187 (1.99%)	61574230 (80.65%)	30785508 (40.32%)	30788722 (40.33%)	40938897 (53.62%)	20635333 (27.03%)
F1	66926392	53935066 (80.59%)	1303116 (1.95%)	52631950 (78.64%)	26330586 (39.34%)	26301364 (39.3%)	34462915 (51.49%)	18169035 (27.15%)

(1) Total mapped represents the total amount of sequencing sequences that can be mapped to the genome. (2) Multiple mapped represents the total amount of sequencing sequences with multiple alignment positions on the reference sequence. (3) Uniquely mapped represents the total amount of sequencing sequences with unique alignment position on the reference sequence. (4) Reads map to ‘+’, Reads map to ‘-’ represent the number of sequencing sequence that aligne to positive and negative chains on the genome

The gene with FPKM > 1 in at least 1 sample was used for analysis (Additional file 3: Table S3). To discover the heterosis in tobacco at the transcriptome level, the differential expression analysis was accomplished by comparing the F1 hybrid to VA116 or Basma, respectively, and also the two parents were compared to each other. At a significant level both of *P* ≤ 0.05 and Fold-change ≥2, 3292 up- and 2612 downregulated transcripts were identified between hybrid and VA116 (Table 3 and Additional file 4: Figure S1A). Similarly, 797 up- and 791 downregulated transcripts were observed between hybrid and Basma (Additional file 4: Figure S1B), and 2951 up- and 2201 downregulated transcripts between Basma and VA116.

**Table 3:** The DEG counts of gene differential expression analyses

Group	Up-regulated DEGs	Down-regulated DEGs	Up-regulated DEGs	Down-regulated DEGs
	(qvalue≤0.05)		(qvalue≤0.05 and FC ≥ 2)	
Basam vs. Va116	3255	3030	2951	2201
F1 vs. Basam	1231	986	797	791
F1 vs. Va116	3888	2908	3292	2612

**Fig. 3:**
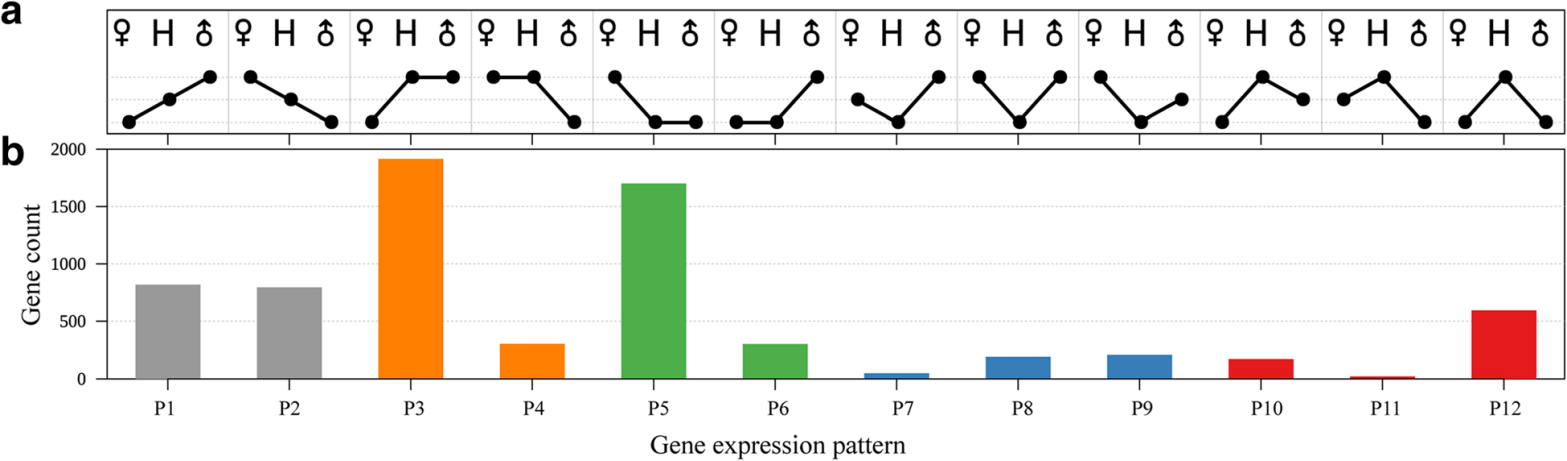
The 12 presumptive additive or non-additive gene expression patterns in F1 hybrid compared to its parents. **a** Expression patterns of 12 types of DEGs. ♂, male parent; H, hybrid; ♀, female parent. **b** A number of genes in each of the 12 types of DEGs

For further analysis of DEGs, the genes were divided into 12 expression patterns (P1–P12, Fig. 3 and Additional file 5: Date S1) as described previously [53]. Genes in P1 and P2 showed an additive expression. Genes in P3–P6 showed a dominant expression, wherein genes in P3 and P4 showed a higher-parent dominance, while P5 and P6 showed a lower-parent dominance. Genes in P7–P12 showed a transgressive expression, wherein genes in P7–P9 showed an up-regulated overdominance, while P10–P12 showed a down-regulated overdominance. In such non-additive expressed genes (P3–P12), genes showing a paternal-expression level dominance (P3 and P5) had the highest proportion.

**Fig. 4:**
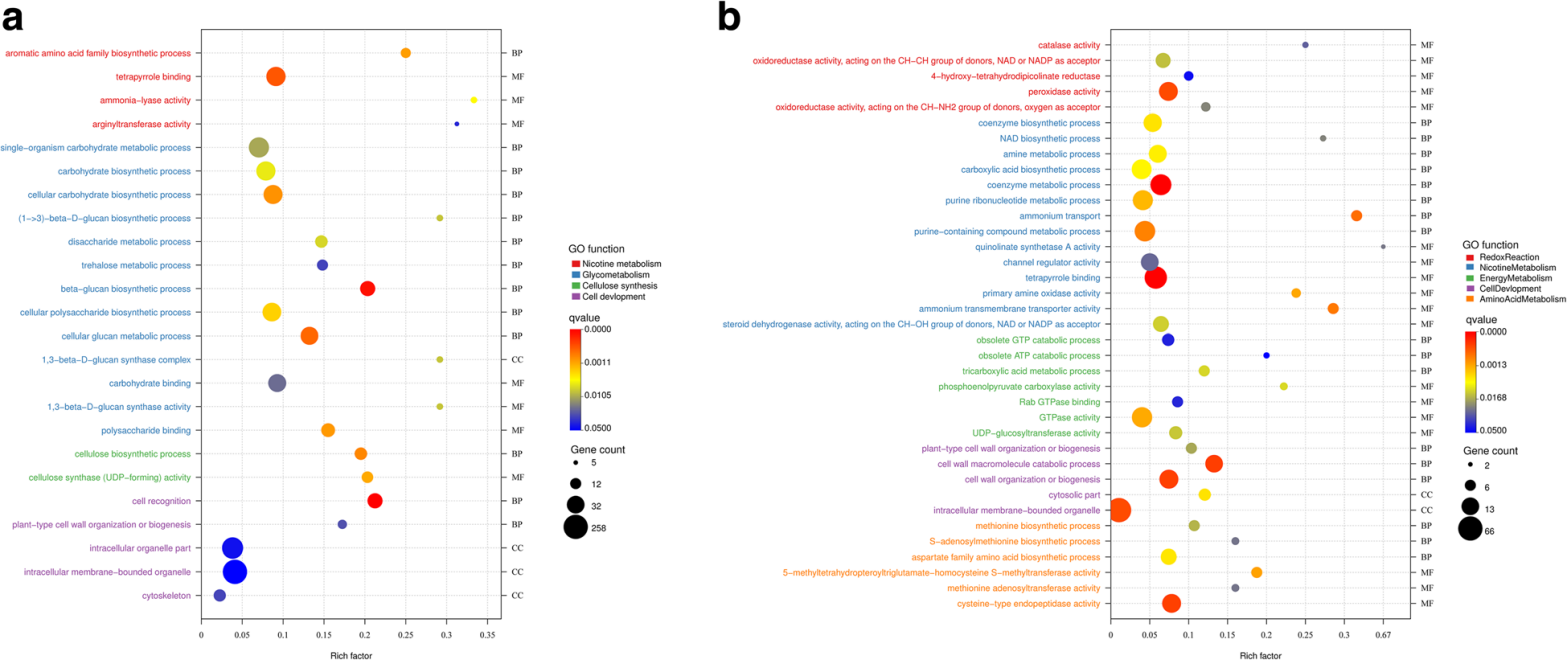
Enriched GO terms for differential gene expression among *Nicotiana tabacum* L. F1 hybrids and its parents. **a** The terms of genes showed a predominant expression in upregulated DEGs. **b** and overdominant expression in upregulated DEGs

To understand the functions of these genes with nonadditive expression, higher-parent dominant genes (HPDGs), lower-parent dominant genes (LPDGs), upregulated overdominant genes (UODGs), and downregulated overdominant genes (DODGs) total 4 gene sets (Additional file 5: Date S1) were respectively implemented for GO and KEGG analysis. The HPDGs were enriched for nicotine metabolism, glycometabolism, cellulose synthesis, and cell development (Fig. 4a). The UODGs were enriched for nicotine metabolism, amino acid metabolism, energy metabolism, redox reaction, and cell wall composition (Fig. 4b). The KEGG analysis the F1 hybrids also showed that a majority of the genes were involved in alkaloid biosynthesis, glycometabolism, phenylpropane metabolic, and vitamin metabolism (Table 4).

**Table 4:** KEGG pathway enrichment of genes showing dominance or transgressive regulation in hybrid

Genetic hypotheses	Regulation pattern	KEGGID	Count	Size	Term	Padjust
Dominance	Down	970	15	24	Aminoacyl-tRNA biosynthesis	0.000828
	UP	940	69	155	Phenylpropanoid biosynthesis	7.87E-07
		909	16	27	Sesquiterpenoid and triterpenoid biosynthesis	0.004545
		360	21	41	Phenylalanine metabolism	0.005072
Overdominance	Down	630	32	80	Glyoxylate and dicarboxylate metabolism	9.57E-16
		30	15	64	Pentose phosphate pathway	0.000399
		51	11	46	Fructose and mannose metabolism	0.003397
		670	5	10	One carbon pool by folate	0.003452
		730	4	7	Thiamine metabolism	0.00693
	UP	960	16	29	Tropane, piperidine and pyridine alkaloid biosynthesis	4.65E-08
		940	36	155	Phenylpropanoid biosynthesis	3.09E-06
		1230	34	174	Biosynthesis of amino acids	0.00028
		531	5	7	Glycosaminoglycan degradation	0.002295
		760	7	15	Nicotinate and nicotinamide metabolism	0.002986
		61	10	33	Fatty acid biosynthesis	0.007455
		945	8	23	Stilbenoid, diarylheptanoid and gingerol biosynthesis	0.009065
		450	6	16	Selenocompound metabolism	0.019903
		460	9	33	Cyanoamino acid metabolism	0.019903
		620	14	67	Pyruvate metabolism	0.020196
		780	5	12	Biotin metabolism	0.022967
		904	4	8	Diterpenoid biosynthesis	0.026009
		270	14	72	Cysteine and methionine metabolism	0.029642
		950	6	19	Isoquinoline alkaloid biosynthesis	0.033853
		750	4	9	Vitamin B6 metabolism	0.036257
		253	3	5	Tetracycline biosynthesis	0.038749
		604	2	2	Glycosphingolipid biosynthesis - ganglio series	0.044164

### Nicotine synthesis genes are significantly altered in the F1 hybrid

**Fig. 5:**
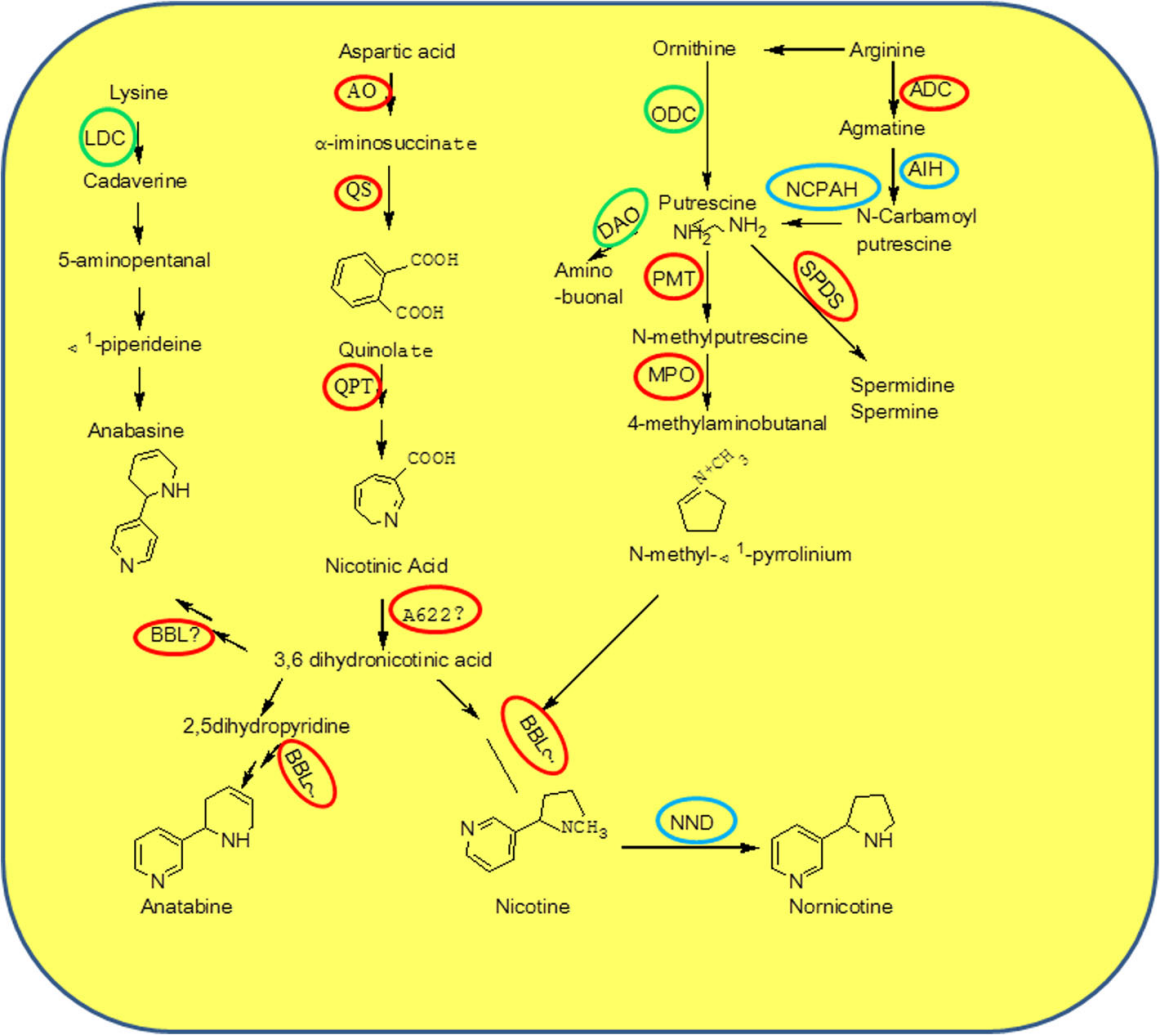
Biosynthetic pathways for nicotine and related metabolites in tobacco. Arrows and double arrows represent enzymatic reactions defined biochemically and undefined steps, respectively. Red, blue, and green circles, represents the gene of overdominant, dominant, and additive expression, respectively

**Fig. 6:**
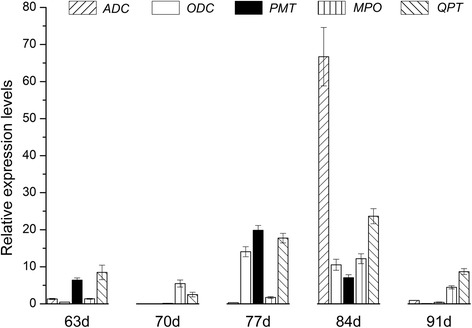
qRT-PCR of DEGs in the nicotine anabolic pathway

**Fig. 7:**
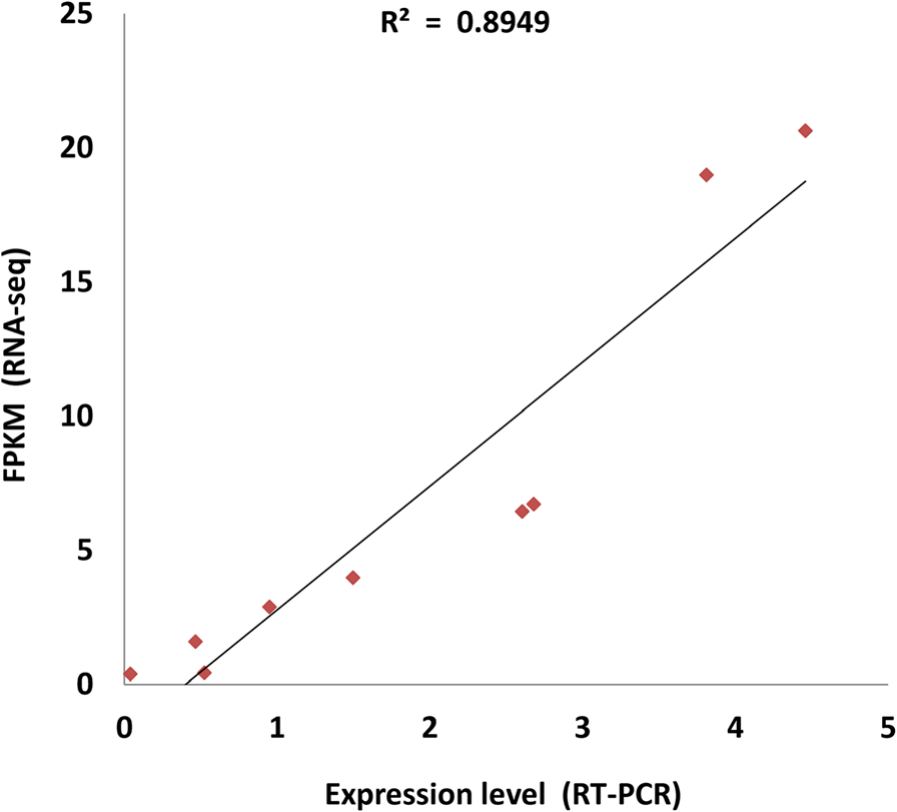
Intercomparison of gene expression values derived from RNA-seq and qRT-PCR. Foldchange was calculated from 11 DEGs, and a high correlation (R2 = 0.89) was obtained between them

From the above, nicotine metabolism was one of the most enriched pathways for the DEGs. As shown in Fig. 5, the genes involved in the formation of the pyridine ring (*AO*, *QS*, *QPT*), and pyrrolidine ring (*ADC*, *PMT*, *MPO*) of nicotine were upregulated in the manner of overdominant expression in the F1 hybrid. The gene expression analysis by RT-PCR confirmed that *ADC, ODC, PMT, MPO,* and *QPT* were upregulated in the hybrid (Fig. 6), and the RNA-seq is highly reliable (Fig. 7). *A622* and *BBLs,* the candidate genes for the pyridine and pyrrolidine rings concatenated in nicotine synthesis; these genes showed upregulated overdominance in the hybrid. *AIH* and *NCPAH* involved in the formation of pyrrole ring, and *NND* in nicotine catabolism showed higher-parent dominant expression. In addition, 5 nicotine transporter genes *JAT2, MATE1 and 2,* and *NUP1 and 2* were upregulated in the manner of overdominant expression in the hybrid; however, the regulatory genes, *ERFs* and *NtMYCs*, for nicotine metabolism showed additive expressions.

## Discussion and conclusions

A previous study showed both interspecific and intraspecific heterosis of nicotine in *nicotina* genus [43, 44]. In the present study, a hybrid, superior for nicotine content, was identified by 3 years of field experiments. Based on the comparison of the transcriptome between of the F1 hybrid and its parents, we discovered substantial transcriptional reprogramming following hybridization, with respectively 4.11% and 12.61% of DEGs displayed changed expression levels in the F1 hybrid. The majority of them were upregulated in the hybrid.

Nicotine anabolism is derived from two independent phases of primary metabolic pathways—the pyridine-nucleotide cycle and the methylpyrrolidine cycle [29, 31]. AO, QS, and QPT participated in the formation of pyridine ring [54, 55], and that ADC, ODC [56], PMT [57] and MPO [58, 59] involved in the pyrrolidine ring synthesis. We found these genes were significantly upregulated in the hybrid except for *ODC*. Notably, the ADC route is preferred for providing the putrescine for nicotine synthesis in the hybrid. A similar conclusion was obtained by using specific inhibitors and C^14^ administration to evaluate the activities of ODC and ADC [29]. However, transgenic experiment suggests that the ODC reaction is primarily responsible for the production of putrescine; nevertheless, ADC plays a minor role in the process [60, 61]. In addition, enzymes encoded by *A622* and *BBLs* genes appear to execute its function in the final steps of nicotine synthesis [32–34], which were upregulated in the F1 hybrid. Taken together, the expression levels of these key genes participating in nicotine synthesis were upregulated in F1 hybrid, which suggest the F1 hybrid has a higher efficiency for nicotine synthesis.

Nicotine is transported from the roots to the vacuoles of leaves by xylem. NUP1 is a plasma membrane-localized transporter, which promotes the import of nicotine from the extracellular medium into the cells [62, 63]. JAT1 [64, 65] and MATE1, 2 [45, 65] were located in the tonoplast of the leaf cells, and they play a major role in nicotine translocation in the aerial parts and deposition in the vacuoles. Therefore, we presumed that the nicotine transport efficiency was also improved in the hybrid F1.

Amino acids are the fundamental units of protein, as well as the original matter for nicotine synthesis. Among the essential amino acids, methionine is a principal metabolite for its functions, not only as a unit for protein synthesis, but also as the precursor of S-adenosylmethionine, polyamines and vitamins [66]. In this study, methionine, S-adenosylmethionine, polyamines and vitamin biosynthetic were induced in F1 hybrid. Aspartic acid, as the precursor of methionine and lysine [67] and the first substance for anabasine, is also the precursor for pyridine ring biosynthesis in nicotine anabolism pathway. In this study, we predict that aspartic acid catabolism was stimulated in F1 hybrid, because it is the precursor of above-mentioned substances.

In addition to nicotine anabolism and amino acid metabolism, other metabolic pathways, such as glycometabolism, cell development, energy metabolism, and redox reaction may also contribute to the nicotine increasing in the hybrids. Nicotine produced in the root undergoes long-distance transport and accumulates mainly in the leaves [68]. Thus, we assumed these pathways involved mainly in the regulation of plant growth, resistance and fitness, which are advantageous for plant development. Superior roots, leaves, xylem formation in the F1 hybrid, source-sink-translocation are all beneficial to the accumulation of nicotine in the leaves. Previously, three quantitative genetic hypotheses had been appointed to explain heterosis: the dominance [1], overdominance [2], and epistasis hypotheses [3]. In this study, a majority of gene for nicotine synthesis and transfer express in a high-parental expression-level dominance pattern suggested that overdominance plays a major role in the heterosis of nicotine.

## Additional files

Additional file 1: **Table S1.** The primer sequences of the randomly selected genes used for RT-PCR validation. (DOCX 16 kb)

Additional file 2: **Table S2.** Quality assessment of sequencing data. (DOCX 13 kb)

Additional file 3: **Table S3.** Number of genes with different expression levels. (DOCX 13 kb)

Additional file 4: **Figure S1.** Volcano plots of significant DEGs in hybrid F1 and its parents in *Nicotiana tabacum* L. The X-axis represents the average value and the Y-axis represents the log_2_ fold-change. Up- or downregulated genes are shown in red (*P* < 0.05) and the others are shown in black (Figure S1A, F1 vs. VA116; Figure S1B, F1 vs. Basma; Figure S1C, VA116 vs. Basma).

Additional file 5: **Date S1.** Significantly enriched GO terms of the DEGs. (XLSX 575 kb)

## Abbreviations

A622: Isoflavone reductase-like protein
ADC: Arginine decarboxylase
AO: Aspartate oxidase
BBL: Berberine bridge enzyme-like
DAO: Diamine oxidase
DEGs: Differentially expressed genes
GO: Gene ontology
GTF: Gene transfer format
KEGG: Kyoto Encyclopedia of Genes and Genomes
MPO: N-methylputrescine oxidase
ODC: Ornithine decarboxylase
PMT: Putrescine methyltransferase
qPCR: Quantitative polymerase chain reaction
QPT: Quinolinate phosphoribosyltransferase
QS: Quinolinate synthase
RT: Reverse transcription
SPDS: Spermidine synthase
