# Supplementary material for: Transcriptomic analysis reveals overdominance playing a critical role in nicotine heterosis in Nicotiana tabacum L
Source: BMC Plant Biol. 2018 Mar 22;18:48. doi: 10.1186/s12870-018-1257-x (PMC5863848; doi:10.1186/s12870-018-1257-x)
Supplement: Supplementary file 1 — Table S1. The primer sequences of the randomly selected genes used for RT-PCR validation. (DOCX 16 kb) [file 12870_2018_1257_MOESM1_ESM.docx]

**Transcriptomic analysis reveals overdominance playing a critical role in Nicotine** **Heterosis** **in** ***Nicotiana tabacum* L.**

Maozhu Tian^1,2^, Qiong Nie^1,2^, Zhenhua Li^1^, Jie Zhang^3^, Yiling Liu^1,2^, Yao Long^1,2^, Zhiwei Wang^1,2^, Guoqing Wang^1,2^, Renxiang Liu^1,2^*

^1^ Key Laboratory of Tobacco Quality in Guizhou province, Guizhou University, Guiyang, 550025, China.

^2^ College of Tobacco, Guizhou University, Guiyang , 550025, China.

^3^ National Maize Improvement Center of China, Beijing Key Laboratory of Crop Genetic Improvement, China Agricultural University, Beijing 100193, China.

*Corresponding author:

Renxiang Liu

Key Laboratory of Tobacco Quality in Guizhou province, Guizhou University, Guiyang , China.

Tel: +86-13985155687

Fax: +86-0851-4117096

E-mail: [rxliu@gzu.edu.cn](mailto:rxliu@gzu.edu.cn)

**Abstract**

**Background:** As a unique biological phenomenon, heterosis has been concerned with the superior performance of the heterosis than either parents. Despite several F1 hybrids, containing supernal nicotine content, had been discovered and applied to heterosis utilization in *Nicotiana tabacum* L., nevertheless, the potential molecular mechanism revealing nicotine heterosis has not been illustrated clearly.

**Result:** Phenotypically, the F1 hybrids (Vall6×Basma) show prominent heterosis in nicotine content by 3 years of field experiments. Transcriptome analysis revealed that genes participating in nicotine anabolism (*ADC, PMT, MPO, QPT,* *AO*, *QS*, *QPT, A622, BBLs*) and nicotine transport (*JAT2, MATE1 and 2, NUP1 and 2*) showed an upregulated expression in the hybrid, a majority of which demonstrated an overdominant performance. RT-PCR confirmed that nicotine anabolism was induced in the hybrid.

**Conclusions:** These ﬁndings strongly suggest that nicotine synthesis and transport efﬁciency improved in hybrid and overdominance at gene-expression level played a critical role in heterosis of nicotine metabolism.

**Keywords**: Nicotine, Heterosis, *Nicotiana tabacum* L., RNA-seq

**1. Background**

Heterosis refers to a unique biological phenomenon that hybrid is superior to either parents in growth potential, yield, resistance, and fitness [1, 2]. Previously, three quantitative genetic hypotheses explained heterosis: the dominance [1], overdominance [2], and epistasis [[3](#_ENREF_1)]. Since then, heterosis is a major concern for both biologists and breeders. Despite the successful agronomic exploitation of heterosis in several crops, especially hybrid rice and hybrid maize, the molecular mechanisms underlying crop heterosis are yet to be elucidated [[4](#_ENREF_2)].

Recently, omics technology has been comprehensively applied in studying the molecular mechanism of heterosis, genome-wide changes in gene expression [[5-9](#_ENREF_3)], siRNA[10], DNA methylation [[11](#_ENREF_9)], and histone modifications [[8](#_ENREF_6), [9](#_ENREF_7)] for hybrids and their parents have been already compared. The genome-wide comparative transcriptional profiling has been exploited to study heterosis in *Arabidopsis* [[12](#_ENREF_10)], rice [[13](#_ENREF_11)], maize [[14](#_ENREF_12)], soybean [[15](#_ENREF_13)], *Brassica napus* [[16](#_ENREF_14)], grape [[17](#_ENREF_15)], petunias [[18](#_ENREF_16)], *Medicago sativa* [[19](#_ENREF_17)], and eucalyptus [[20](#_ENREF_18)]. Using genome-wide association studies (GWAS) approach, several superior alleles that contributed to yield heterosis were identified in rice [[21](#_ENREF_19)], significant locus and candidate genes that contributed to flowering period and biomass-related heterosis were verified in *Arabidopsis* [[22](#_ENREF_20), [23](#_ENREF_21)]. In addition, the mapping for heterosis quantitative trait loci on yield in rice hybrids has been reported [[4](#_ENREF_2), [24](#_ENREF_22)]. However, none of studies have investigated heterosis in *Nicotiana tabacum* L., which is an important model species in Solanaceae.

Nicotine, a main alkaloid in *Nicotiana tabacum* L., played a pivotal role in *Solanaceae* plant defense against diseases and pests. Accompanied by biosynthesis in roots [[25](#_ENREF_23)], nicotine is translocated into leaf by xylem transport [[26](#_ENREF_24)]. Nicotine is synthesized by two different nitrogen-containing rings, the pyrrole ring and the pyridine ring, which are derived from different synthetic pathways [[27](#_ENREF_25)]. For pyrrole ring, nicotine biosynthesis could initiate directly through the decarboxylation of ornithine by ODC to form putrescine [[28](#_ENREF_26)] or indirectly through ADC-mediated decarboxylation of arginine to form putrescine [[29](#_ENREF_27)]. Then, putrescine is catalyzed by PMT, a key regulatory enzyme for nicotine anabolism, to form N-methylputrescine [[30](#_ENREF_28)]. Finally, N-methylputrescine is catalyzed by MPO, forming the direct precursor of nicotine. For pyridine ring, QPT is the rate-limiting enzyme in nicotine anabolism [[31](#_ENREF_29)]. Recently, enzymes encoded by genes *A622* and *BBLs*, were deduced to function in the last steps of nicotine synthesis [[32-34](#_ENREF_30)].

*Nicotiana tabacum* L. is a classic allotetraploid crop species developed by natural hybridization of two progenitor species, *Nicotiana sylvestris* and *Nicotiana tomentosiformis* [[35](#_ENREF_33)], interspecific and intraspecific of *nicotina* genus all had sufficient amount of heterosis [[36-39](#_ENREF_34)]. Heterosis has been widely studied with respect to growth rate, flower days, plant height, and leaf yield [40-42]. Reportedly, some hybrids in flue-cured varieties, between *N. rustica* and *N. tabacum*, showed a positive heterosis in the nicotine content [43, 44]. However, molecular mechanisms of nicotine heterosis have not been illustrated in tobacco. Hence, this study aimed to reveal it by transcriptome analysis using model plant *Nicotiana tabacum* L. To our knowledge, this is the first study to describe nicotine heterosis in plants on a transcriptomic scale.

**2.** **Materials and Methods**

**2.1 Plant materials and nicotine analysis**

Tobacco hybrid F1, derived by crossing the varieties of Va116 (female parent, *flue-cured* *tobacco*) and Basma (male parent, Oriental tobacco). Va116 and Basma were collected from Tobacco Institute at Chinese Academy of Agriculture Sciences (CAAS). The seeds were sowed in floating plates and grown in a greenhouse until the seedlings contains five euphylla. Using a randomized block design, all seedlings were planted with three replicates, with planting distance and space of 110 × 55 cm on the experimental base at Guizhou University in 2013, 2014, and 2015, and it must be stated that they were topped 70 days after transplanting. The leaves and roots were collected at 7-day intervals from 56 to 91 days after transplantation. All leaves and roots samples were collected as follows: at least 10 of them were picked from the mother plants, respectively, mixed as a biological repetition, and three repetitions were used for the experiment. Leaf nicotine were obtained from the extract of dry samples and measured by gas chromatography according to described by Shoji T. et al [[45](#_ENREF_38)]. In addition, root samples were collected in 2015 for genes expression analysis (7-day intervals from 63 to 91 days after transplantation) and transcriptomics (samples collected 77 days after transplantation).

**2.2** **RNA isolation and sequencing**

The root tissues were treated with Total RNA purification Kit (LC Science, TRK-1001) to extract the total RNA, the whole process was done at the manufacturer's suggested protocols. All the total RNA samples were controlled in a high quality condition, in which A260/A280 >2, RIN value >7. Subsequently, the mRNA was puriﬁed from 5 µg total RNA by poly-T oligo-attached magnetic beads, to obtain fragments of ~100–400 bp. The RNA fragments were used for the first strand-cDNA synthesis by using reverse transcriptase and random primers. Followed, second-strand cDNA was synthesized by using DNA polymerase I and RNase H. Agilent 2100 Bioanalyzer and ABI StepOne Plus Real-Time PCR System were used for the qualitative and quantitative analyses of all libraries. Three cDNA libraries, with 200 bp insert size, were selected for sequencing with the Illumina HiSeq 2500 platform (Illumina Inc., San Diego, CA, USA).

**2.3** **Transcriptomics data processing and analysis**

The raw reads were preprocessed. In this process, the adaptor sequences, short sequences with a length <25 nt, low quality sequences were removed. After preprocessing, the obtained reads were mapped to the *Nicotiana tabacum* sequenced cultivar K326 genome using the splice-aware mapping tool, Tophat2 [[46](#_ENREF_39)]. The intermediate result files generated by Tophat2 were used as the input data sets of Cufflinks [[47](#_ENREF_40)]. Subsequently, the libraries, that were assembled by Cufflinks and Cuffmerge [[47](#_ENREF_40)], were used to merge these assemblies in order to find the novel expressed gene loci. Then, the abundance in expression (FPKM) of all genes was estimated by Cufflinks. The novel expressed genes with longest transcript ≥200bp were annotated using the NCBI NR database <<ftp://ftp.ncbi.nih.gov/blast/db>> and KEGG pathway (www.genome.jp). The differential expression genes (DEG) were analyzed using the statistical tool R and DESeq package [[48](#_ENREF_41)]. The P-values of DEGs were corrected by Benjamini–Hochberg FDR (false discovery rate) correction [[49](#_ENREF_42)]. The over-representation of GO terms and KEGG pathways of DEGs were identified using the Goatools (https://github.com/tanghaibao/Goatools) and R GO package [[50](#_ENREF_43)] respectively. The P-value of the enriched analyses was corrected by the FDR method.

**2.4** **Real-time PCR**

To verify the expression levels of tobacco genes obtained from RNA sequencing, Real-time PCR (RT-qPCR) experiments were conducted to quantify the expression of a few random genes. Quantitative Real-time PCR was performed using SYBR Premix Ex Taq Kit (Takara) according to the manufacturer’s protocol. The RT-qPCR reactions were conducted on Applied Biosystems 7500 Real-Time PCR System (Life Technologies Corporation, Beverly, MA, USA). Two Actin genes were used as the endogenous reference genes, and the expression level of each gene was normalized against the Actin-2 gene. Genes and primers for the qPCR are listed in Supplemental Table S1. To calculate the relative expression level of individual gene, 2^−ΔΔ Ct^ method [[44](#_ENREF_44)] was adopted. The expression data of all genes, which had being generated by the real-time PCR experiments, were displayed as average values with standard error appended.

**2.5 Statistical analysis**

Field experiment design was randomized with three replicates (see “Plant materials and nicotine analysis” part). The nicotine content was used for ANOVA, which was performed using Duncan’s test in SPSS software Ver.16.0. (P < 0.05). Over high-parent heterosis (OPH), mid-parent heterosis (MPH), and below low-parent heterosis (BPH) was calculated as follows: OPH (%) =$(\frac{F1-HP}{\mathrm{HP}})\times100$, MPH (%) =$(\frac{F1-MP}{\mathrm{MP}})\times100$, BPH (%) =$(\frac{F1-LP}{\mathrm{LP}})\times100$, where; F1 = performance of the hybrid, HP = high-value parent, MP = mid-parent average$\left[ \frac{\left( parent1+parent2 \right)}{2} \right],$

LP = low-value parent.

**3. Results**

**3.1 Nicotine content significantly increased in roots of F1 hybrids**

The nicotine content of parental and its hybrid was measured from 56–91 days after transplant, and the results were summarized in Fig. 1. During development, a rapid increase in the nicotine content was observed during the initial 3 weeks (56–77 days, especially 70-77 days) with both parents and its hybrid, while a gradual increase was observed subsequently (77–91 days). The growth rate of nicotine content for the hybrid exceeded that of the parents. As shown in Fig. 2, continuous 3 years of the experimental study revealed that the nicotine content of the hybrids was significantly higher than that of parents after topping. However, no significant difference was observed between them before topping. Table 1 displayed less difference in OPH, MPH, and BPH heterosis values before topping; conversely, these were increased significantly after topping. The mid-parent (MPH) value (MPV) was ~40% higher.

**3.2 Transcriptome differences of leaves between parents and F1 hybrids**

RNA sequencing technique was employed to study the nicotine heterosis of tobacco. Three paired-end libraries, VA116, Basma, and their hybrid, were constructed and sequenced on the Illumina HiSeq 2000 platform. The quality assessment of the sequencing data was shown in Table S1; clean reads accounted for >95% of the total reads with error rates of 0.02%, Q20 > 96%, Q30 > 91%. The read counts and genomic mapping parameters of the three libraries are listed in Table S1, which showed that 80.59–82.64% clean reads were mapped on the *N. tabacum* L. K326 reference genome [[52](#_ENREF_45)].

The gene with FPKM >1 in at least 1 sample was used for analysis (Table S2). To discover the heterosis in tobacco at the transcriptome level, the differential expression analysis was accomplished by comparing the F1 hybrid to VA116 or Basma, respectively, and also the two parents were compared to each other. At a significant level both of P ≤ 0.05 and Fold-change ≥ 2, 3292 up- and 2612 downregulated transcripts were identified between hybrid and VA116 (Table 3 and Fig. S1A). Similarly, 797 up- and 791 downregulated transcripts were observed between hybrid and Basma (Fig. S1B), and 2951 up- and 2201 downregulated transcripts between Basma and VA116.

For further analysis of DEGs, the genes were divided into 12 expression patterns (P1–P12, Fig. 3 and date S1) as described previously [[53](#_ENREF_46)]. Genes in P1 and P2 showed an additive expression. Genes in P3–P6 showed a dominant expression, wherein genes in P3 and P4 showed a higher-parent dominance, while P5 and P6 showed a lower-parent dominance. Genes in P7–P12 showed a transgressive expression, wherein genes in P7–P9 showed an up-regulated overdominance, while P10–P12 showed a down-regulated overdominance. In such non-additive expressed genes (P3–P12), genes showing a paternal-expression level dominance (P3 and P5) had the highest proportion.

To understand the functions of these genes with nonadditive expression, higher-parent dominant genes (HPDGs), lower-parent dominant genes (LPDGs), upregulated overdominant genes (UODGs), and downregulated overdominant genes (DODGs) total 4 gene sets (Date S1) were respectively implemented for GO and KEGG analysis. The HPDGs were enriched for nicotine metabolism, glycometabolism, cellulose synthesis, and cell development (Fig. 4A). The UODGs were enriched for nicotine metabolism, amino acid metabolism, energy metabolism, redox reaction, and cell wall composition (Fig. 4B). The KEGG analysis the F1 hybrids also showed that a majority of the genes were involved in alkaloid biosynthesis, glycometabolism, phenylpropane metabolic, and vitamin metabolism (Table 4).

**3.3 Nicotine synthesis genes are signiﬁcantly altered in the F1 hybrid**

From the above, nicotine metabolism was one of the most enriched pathways for the DEGs. As shown in Fig. 5, the genes involved in the formation of the pyridine ring (*AO*, *QS*, *QPT*), and pyrrolidine ring (*ADC*, *PMT*, *MPO*) of nicotine were upregulated in the manner of overdominant expression in the F1 hybrid. The gene expression analysis by RT-PCR confirmed that *ADC, ODC, PMT, MPO,* and *QPT* were upregulated in the hybrid (Fig. 6), and the RNA-seq is highly reliable (Fig. 7). *A622* and *BBLs,* the candidate genes for the pyridine and pyrrolidine rings concatenated in nicotine synthesis; these genes showed upregulated overdominance in the hybrid. *AIH* and *NCPAH* involved in the formation of pyrrole ring, and *NND* in nicotine catabolism showed higher-parent dominant expression. In addition, 5 nicotine transporter genes *JAT2, MATE1 and 2,* and *NUP1 and 2* were upregulated in the manner of overdominant expression in the hybrid; however, the regulatory genes, *ERFs* and *NtMYCs*, for nicotine metabolism showed additive expressions.

**4. Discussion**

A previous study showed both interspecific and intraspecific heterosis of nicotine in *nicotina* genus [43, 44]. In the present study, a hybrid, superior for nicotine content, was identified by three years of field experiments. Based on the comparison of the transcriptome between of the F1 hybrid and its parents, we discovered substantial transcriptional reprogramming following hybridization, with respectively 4.11% and 12.61% of DEGs displayed changed expression levels in the F1 hybrid. The majority of them were upregulated in the hybrid.

Nicotine anabolism is derived from two independent phases of primary metabolic pathways—the pyridine-nucleotide cycle and the methylpyrrolidine cycle [[29](#_ENREF_27), [31](#_ENREF_29)]. AO, QS, and QPT participated in the formation of pyridine ring [[54](#_ENREF_47), [55](#_ENREF_48)], and that ADC, ODC [[56](#_ENREF_49)], PMT [[57](#_ENREF_50)] and MPO [[58](#_ENREF_51), [59](#_ENREF_52)] involved in the pyrrolidine ring synthesis. We found these genes were signiﬁcantly upregulated in the hybrid except for *ODC*. Notably, the ADC route is preferred for providing the putrescine for nicotine synthesis in the hybrid. A similar conclusion was obtained by using specific inhibitors and C^14^ administration to evaluate the activities of ODC and ADC [[29](#_ENREF_27)]. However, transgenic experiment suggests that the ODC reaction is primarily responsible for the production of putrescine; nevertheless, ADC plays a minor role in the process [[60](#_ENREF_53), [61](#_ENREF_54)]. In addition, enzymes encoded by *A622* and *BBLs* genes appear to execute its function in the final steps of nicotine synthesis [[32-34](#_ENREF_30)], which were upregulated in the F1 hybrid. Taken together, the expression levels of these key genes participating in nicotine synthesis were upregulated in F1 hybrid, which suggest the F1 hybrid has a higher efficiency for nicotine synthesis.

Nicotine is transported from the roots to the vacuoles of leaves by xylem. NUP1 is a plasma membrane-localized transporter, which promotes the import of nicotine from the extracellular medium into the cells [[62](#_ENREF_55), [63](#_ENREF_56)]. JAT1 [[64](#_ENREF_57), [65](#_ENREF_58)] and MATE1, 2 [[45](#_ENREF_38), [65](#_ENREF_58)] were located in the tonoplast of the leaf cells, and they play a major role in nicotine translocation in the aerial parts and deposition in the vacuoles. Therefore, we presumed that the nicotine transport efﬁciency was also improved in the hybrid F1.

Amino acids are the fundamental units of protein, as well as the original matter for nicotine synthesis. Among the essential amino acids, methionine is a principal metabolite for its functions, not only as a unit for protein synthesis, but also as the precursor of S-adenosylmethionine, polyamines and vitamins [[66](#_ENREF_59)]. In this study, methionine, S-adenosylmethionine, polyamines and vitamin biosynthetic were induced in F1 hybrid. Aspartic acid, as the precursor of methionine and lysine [[67](#_ENREF_60)] and the first substance for anabasine, is also the precursor for pyridine ring biosynthesis in nicotine anabolism pathway. In this study, we predict that aspartic acid catabolism was stimulated in F1 hybrid, because it is the precursor of above-mentioned substances.

In addition to nicotine anabolism and amino acid metabolism, other metabolic pathways, such as glycometabolism, cell development, energy metabolism, and redox reaction may also contribute to the nicotine increasing in the hybrids. Nicotine produced in the root undergoes long-distance transport and accumulates mainly in the leaves [[68](#_ENREF_61)]. Thus, we assumed these pathways involved mainly in the regulation of plant growth, resistance and fitness, which are advantageous for plant development. Superior roots, leaves, xylem formation in the F1 hybrid, source-sink-translocation are all beneficial to the accumulation of nicotine in the leaves. Previously, three quantitative genetic hypotheses had been appointed to explain heterosis: the dominance [1], overdominance [2], and epistasis hypotheses [[3](#_ENREF_1)]. In this study, a majority of gene for nicotine synthesis and transfer express in a high-parental expression-level dominance pattern suggested that overdominance plays a major role in the heterosis of nicotine.

**Figure legends**

**Figure 1.** Nicotine content of F1 hybrids and their parents during leaf development.

**Figure 2.** Heterosis of the F1 hybrid in *Nicotiana tabacum* L. of the two developmental stages, BT and AT, which represent before topping and after topping, respectively.

**Figure 3.** The 12 presumptive additive or non-additive gene expression patterns in F1 hybrid compared to its parents. (a) Expression patterns of 12 types of DEGs. ♂, male parent; H, hybrid; ♀, female parent. (b) A number of genes in each of the 12 types of DEGs.

**Figure 4.** Enriched GO terms for differential gene expression among *Nicotiana tabacum* L. F1 hybrids and its parents. (A) The terms of genes showed a predominant expression in upregulated DEGs. (B) and overdominant expression in upregulated DEGs.

**Figure 5.** Biosynthetic pathways for nicotine and related metabolites in tobacco. Arrows and double arrows represent enzymatic reactions defined biochemically and undefined steps, respectively. Red, blue, and green circles, represents the gene of overdominant, dominant, and additive expression, respectively.

**Figure 6.** Intercomparison of gene expression values derived from RNA-seq and qRT-PCR. Foldchange was calculated from 11 DEGs, and a high correlation (R^2^ = 0.89) was obtained between them.

**Additional files**

**Additional file 1:** **Figure S1.** Volcano plots of significant DEGs in hybrid F1 and its parents in *Nicotiana tabacum* L. The X-axis represents the average value and the Y-axis represents the log_2_ fold-change. Up- or downregulated genes are shown in red (P < 0.05) and the others are shown in black (Fig. S1A, F1 vs. VA116; Fig. S1B, F1 vs. Basma; Fig. S1C, VA116 vs. Basma).

Additional file 2: Table S1. The primer sequences of the randomly selected genes used for RT-PCR validation

Additional file 3: Table S2 Quality assessment of sequencing data

Additional file 4: Table S3 Number of genes with different expression levels

Additional file 5: Date S1. Significantly enriched GO terms of the DEGs

**Abbreviations**

AO, aspartate oxidase

BBL, berberine bridge enzyme-like

DAO, diamine oxidase

ADC, arginine decarboxylase

ODC, ornithine decarboxylase

PMT, putrescine methyltransferase

MPO, N-methylputrescine oxidase

QPT, quinolinate phosphoribosyltransferase

GO, Gene Ontology

KEGG, Kyoto Encyclopedia of Genes and Genomes

GTF, gene transfer format

qPCR, quantitative polymerase chain reaction

RT, reverse transcription

DEGs, differentially expressed genes

SPDS, spermidine synthase

QS, quinolinate synthase

A622, isoflavone reductase-like protein

**Acknowledgements**

None

**Authors’ contributions**

TMZ, LYL and LY performed most of the field work; TMZ, NQ, WGQ, WZW and LYL carried out the molecular biology and chemistry studies. ZJ and LZH analyzed the transcriptome data. LZH, TMZ, and LRX drafted the manuscript. LRX and LZH conceived the study and participated in the design and coordination. All authors have read and approved the final manuscript. **Funding**

This research was financially supported by the Talent and Team Cultivation Project for High-Level Renovation Plan of Guizhou Province ([2015]4011 and 20165663) and Key projects from the Guizhou Provincial Tobacco Company of China National Tobacco Corporation (201302 and 201602).The funders made no significant role in study design, data collection and analysis, decision to publish, or writing of the manuscript.

**Availability of data and materials**

The materials used during the current study will be freely available upon request to corresponding author: rxliu@gzu.edu.cn for reasonable use only. RNA-seq read date has been deposited in the NCBI datebase undrer the accession number PRJNA432797.

**Ethics approval and consent to participate**

The seeds of VA116 and Basma were collected from Tobacco Institute of Chinese Academy of Agriculture Sciences (CAAS).

**Consent for publish**

Not applicable

**Competing interests**

The authors declare that they have no competing interests.

**References**

1. Shull GH. 1908. The composition of a field of maize. Journal of Heredity 4, 296–301

2. Bruce AB. 1910. The Mendelian theory of heredity and the augmentation of vigor. Science 32, 627–628.

3. Schnell FW, Cockerham CC: Multiplicative vs. arbitrary gene action in heterosis. Genetics 1992, 131(2):461-469.

4. Wei G, Tao Y, Liu G, Chen C, Luo R, Xia H, Gan Q, Zeng H, Lu Z, Han Y et al: A transcriptomic analysis of superhybrid rice LYP9 and its parents. Proceedings of the National Academy of Sciences of the United States of America 2009, 106(19):7695-7701.

5. Groszmann M, Gonzalez-Bayon R, Lyons RL, Greaves IK, Kazan K, Peacock WJ, Dennis ES: Hormone-regulated defense and stress response networks contribute to heterosis in Arabidopsis F1 hybrids. Proceedings of the National Academy of Sciences of the United States of America 2015, 112(46):E6397-6406.

6. Fujimoto R, Taylor JM, Shirasawa S, Peacock WJ, Dennis ES: Heterosis of Arabidopsis hybrids between C24 and Col is associated with increased photosynthesis capacity. Proceedings of the National Academy of Sciences of the United States of America 2012, 109(18):7109-7114.

7. Miller M, Song Q, Shi X, Juenger TE, Chen ZJ: Natural variation in timing of stress-responsive gene expression predicts heterosis in intraspecific hybrids of Arabidopsis. Nature communications 2015, 6:7453.

8. He G, Zhu X, Elling AA, Chen L, Wang X, Guo L, Liang M, He H, Zhang H, Chen F et al: Global epigenetic and transcriptional trends among two rice subspecies and their reciprocal hybrids. The Plant cell 2010, 22(1):17-33.

9. He G, Chen B, Wang X, Li X, Li J, He H, Yang M, Lu L, Qi Y, Wang X et al: Conservation and divergence of transcriptomic and epigenomic variation in maize hybrids. Genome biology 2013, 14(6):R57.

10. Groszmann M, Greaves IK, Albertyn ZI, Scofield GN, Peacock WJ, Dennis ES: Changes in 24-nt siRNA levels in Arabidopsis hybrids suggest an epigenetic contribution to hybrid vigor. Proceedings of the National Academy of Sciences of the United States of America 2011, 108(6):2617-2622.

11. Shen H, He H, Li J, Chen W, Wang X, Guo L, Peng Z, He G, Zhong S, Qi Y et al: Genome-wide analysis of DNA methylation and gene expression changes in two Arabidopsis ecotypes and their reciprocal hybrids. The Plant cell 2012, 24(3):875-892.

12. Wang J, Tian L, Lee HS, Wei NE, Jiang H, Watson B, Madlung A, Osborn TC, Doerge RW, Comai L et al: Genomewide nonadditive gene regulation in Arabidopsis allotetraploids. Genetics 2006, 172(1):507-517.

13. Swanson-Wagner RA, Jia Y, DeCook R, Borsuk LA, Nettleton D, Schnable PS: All possible modes of gene action are observed in a global comparison of gene expression in a maize F1 hybrid and its inbred parents. Proceedings of the National Academy of Sciences of the United States of America 2006, 103(18):6805-6810.

14. Huang Y, Zhang L, Zhang J, Yuan D, Xu C, Li X, Zhou D, Wang S, Zhang Q: Heterosis and polymorphisms of gene expression in an elite rice hybrid as revealed by a microarray analysis of 9198 unique ESTs. Plant molecular biology 2006, 62(4-5):579-591.

15. Zhang C, Lin C, Fu F, Zhong X, Peng B, Yan H, Zhang J, Zhang W, Wang P, Ding X et al: Comparative transcriptome analysis of flower heterosis in two soybean F1 hybrids by RNA-seq. PloS one 2017, 12(7):e0181061.

16. Renaut S, Rowe HC, Ungerer MC, Rieseberg LH: Genomics of homoploid hybrid speciation: diversity and transcriptional activity of long terminal repeat retrotransposons in hybrid sunflowers. Philosophical transactions of the Royal Society of London Series B, Biological sciences 2014, 369(1648).

17. Wang L, Hu X, Jiao C, Li Z, Fei Z, Yan X, Liu C, Wang Y, Wang X: Transcriptome analyses of seed development in grape hybrids reveals a possible mechanism influencing seed size. BMC genomics 2016, 17(1):898.

18. Zhuang Y, Tripp EA: Genome-scale transcriptional study of hybrid effects and regulatory divergence in an F1 hybrid Ruellia (Wild Petunias: Acanthaceae) and its parents. BMC plant biology 2017, 17(1):15.

19. Li X, Wei Y, Nettleton D, Brummer EC: Comparative gene expression profiles between heterotic and non-heterotic hybrids of tetraploid Medicago sativa. BMC plant biology 2009, 9:107.

20. Shinya T, Iwata E, Nakahama K, Fukuda Y, Hayashi K, Nanto K, Rosa AC, Kawaoka A: Transcriptional Profiles of Hybrid Eucalyptus Genotypes with Contrasting Lignin Content Reveal That Monolignol Biosynthesis-related Genes Regulate Wood Composition. Frontiers in plant science 2016, 7:443.

21. Huang X, Yang S, Gong J, Zhao Y, Feng Q, Gong H, Li W, Zhan Q, Cheng B, Xia J et al: Genomic analysis of hybrid rice varieties reveals numerous superior alleles that contribute to heterosis. Nature communications 2015, 6:6258.

22. Seymour DK, Chae E, Grimm DG, Martin Pizarro C, Habring-Muller A, Vasseur F, Rakitsch B, Borgwardt KM, Koenig D, Weigel D: Genetic architecture of nonadditive inheritance in Arabidopsis thaliana hybrids. Proceedings of the National Academy of Sciences of the United States of America 2016, 113(46):E7317-E7326.

23. Yang M, Wang X, Ren D, Huang H, Xu M, He G, Deng XW: Genomic architecture of biomass heterosis in Arabidopsis. Proceedings of the National Academy of Sciences of the United States of America 2017.

24. Huang X, Yang S, Gong J, Zhao Q, Feng Q, Zhan Q, Zhao Y, Li W, Cheng B, Xia J et al: Genomic architecture of heterosis for yield traits in rice. Nature 2016, 537(7622):629-633.

25. Dawson RF, Solt ML: Estimated Contributions of Root and Shoot to the Nicotine Content of the Tobacco Plant. Plant physiology 1959, 34(6):656-661.

26. Tso TC, Jeffrey RN: Studies on Tobacco Alkaloids. II. The Formation of Nicotine and Nornicotine in Tobacco Supplied with N. Plant physiology 1957, 32(2):86-92.

27. Hakkinen ST, Tilleman S, Swiatek A, De Sutter V, Rischer H, Vanhoutte I, Van Onckelen H, Hilson P, Inze D, Oksman-Caldentey KM et al: Functional characterisation of genes involved in pyridine alkaloid biosynthesis in tobacco. Phytochemistry 2007, 68(22-24):2773-2785.

28. Marton LJ, Pegg AE: Polyamines as targets for therapeutic intervention. Annual review of pharmacology and toxicology 1995, 35:55-91.

29. Tiburcio AF, Galston AW: Arginine decarboxylase as the source of putrescine for tobacco alkaloids. Phytochemistry 1986, 25(1):107-110.

30. Saunders JW, Bush LP: Nicotine Biosynthetic Enzyme Activities in Nicotiana tabacum L. Genotypes with Different Alkaloid Levels. Plant physiology 1979, 64(2):236-240.

31. Wagner R, Feth F, Wagner KG: Regulation in tobacco callus of enzyme activities of the nicotine pathway : II. The pyridine-nucleotide cycle. Planta 1986, 168(3):408-413.

32. Deboer KD, Lye JC, Aitken CD, Su AK, Hamill JD: The A622 gene in Nicotiana glauca (tree tobacco): evidence for a functional role in pyridine alkaloid synthesis. Plant molecular biology 2009, 69(3):299-312.

33. Kajikawa M, Hirai N, Hashimoto T: A PIP-family protein is required for biosynthesis of tobacco alkaloids. Plant molecular biology 2009, 69(3):287-298.

34. Kajikawa M, Shoji T, Kato A, Hashimoto T: Vacuole-localized berberine bridge enzyme-like proteins are required for a late step of nicotine biosynthesis in tobacco. Plant physiology 2011, 155(4):2010-2022.

35. Murad L, Lim KY, Christopodulou V, Matyasek R, Lichtenstein CP, Kovarik A, Leitch AR: The origin of tobacco's T genome is traced to a particular lineage within Nicotiana tomentosiformis (Solanaceae). American journal of botany 2002, 89(6):921-928.

36. Evans DA, Flick CE, Kut SA, Reed SM: Comparison of Nicotiana tabacum and Nicotiana nesophila hybrids produced by ovule culture and protoplast fusion. TAG Theoretical and applied genetics Theoretische und angewandte Genetik 1982, 62(3):193-198.

37. Xu ZC, Zhu J: An approach for predicting heterosis based on an additive, dominance and additive x additive model with environment interaction. Heredity 1999, 82 ( Pt 5):510-517.

38. Trojak-Goluch A, Berbec A: Cytological investigations of the interspecific hybrids of Nicotiana tabacum L. x N. glauca Grah. Journal of applied genetics 2003, 44(1):45-54.

39. Tezuka T, Marubashi W: Genes in S and T subgenomes are responsible for hybrid lethality in interspecific hybrids between Nicotiana tabacum and Nicotiana occidentalis. PloS one 2012, 7(4):e36204.

40 Chaplin JF Comparative performance of F1 flue cured tobacco hybrids and their parents. I. Agronomic and quality characteristics. Tob science 1966, 10:126-130

41 DeanCEHeterosis, inbreedingdepression,and combining ability in diallel crosses of cigar-

wrapper tobacco. Crop science 1974, 14:482-482

42 Hancock W G, Lewis R S. Heterosis, transmission genetics, and selection for increased growth rate in a N. tabacum × synthetic tobacco cross. Mol Breeding 2017, 37: 53,1-19

43 Sabour M, Simmoneisi J, setterfieed G. Variation in nicotine content of cultured cell lines of nicotians species and their somatic and sexual hybrids. Plant Breeding, 1986,324-333.

44 Vandenberg P, Matzinger DF. Genetic diversity and heterosis in Nicotiana. III. Crosses among tobacco introductions and flue-cured varieties. Crop science 1970, 10:437-440

45. Shoji T, Inai K, Yazaki Y, Sato Y, Takase H, Shitan N, Yazaki K, Goto Y, Toyooka K, Matsuoka K et al: Multidrug and toxic compound extrusion-type transporters implicated in vacuolar sequestration of nicotine in tobacco roots. Plant physiology 2009, 149(2):708-718.

46. Kim D, Pertea G, Trapnell C, Pimentel H, Kelley R, Salzberg SL: TopHat2: accurate alignment of transcriptomes in the presence of insertions, deletions and gene fusions. Genome biology 2013, 14(4):R36.

47. Trapnell C, Williams BA, Pertea G, Mortazavi A, Kwan G, van Baren MJ, Salzberg SL, Wold BJ, Pachter L: Transcript assembly and quantification by RNA-Seq reveals unannotated transcripts and isoform switching during cell differentiation. Nature biotechnology 2010, 28(5):511-515.

48. Anders S, Huber W: Differential expression analysis for sequence count data. Genome biology 2010, 11(10):R106.

49. Nelson DE, Rammesmayer G, Bohnert HJ: Regulation of cell-specific inositol metabolism and transport in plant salinity tolerance. The Plant cell 1998, 10(5):753-764.

50. Falcon S, Gentleman R: Using GOstats to test gene lists for GO term association. Bioinformatics 2007, 23(2):257-258.

51. Livak KJ, Schmittgen TD: Analysis of relative gene expression data using real-time quantitative PCR and the 2(-Delta Delta C(T)) Method. Methods 2001, 25(4):402-408.

52. Sierro N, Battey JN, Ouadi S, Bakaher N, Bovet L, Willig A, Goepfert S, Peitsch MC, Ivanov NV: The tobacco genome sequence and its comparison with those of tomato and potato. Nature communications 2014, 5:3833.

53. Yoo MJ, Szadkowski E, Wendel JF: Homoeolog expression bias and expression level dominance in allopolyploid cotton. Heredity 2013, 110(2):171-180.

54. Sinclair SJ, Murphy KJ, Birch CD, Hamill JD: Molecular characterization of quinolinate phosphoribosyltransferase (QPRtase) in Nicotiana. Plant molecular biology 2000, 44(5):603-617.

55. Katoh A, Uenohara K, Akita M, Hashimoto T: Early steps in the biosynthesis of NAD in Arabidopsis start with aspartate and occur in the plastid. Plant physiology 2006, 141(3):851-857.

56. Imanishi S, Hashizume K, Nakakita M, Kojima H, Matsubayashi Y, Hashimoto T, Sakagami Y, Yamada Y, Nakamura K: Differential induction by methyl jasmonate of genes encoding ornithine decarboxylase and other enzymes involved in nicotine biosynthesis in tobacco cell cultures. Plant molecular biology 1998, 38(6):1101-1111.

57. Hibi N, Higashiguchi S, Hashimoto T, Yamada Y: Gene expression in tobacco low-nicotine mutants. The Plant cell 1994, 6(5):723-735.

58. Heim WG, Sykes KA, Hildreth SB, Sun J, Lu RH, Jelesko JG: Cloning and characterization of a Nicotiana tabacum methylputrescine oxidase transcript. Phytochemistry 2007, 68(4):454-463.

59. Katoh A, Shoji T, Hashimoto T: Molecular cloning of N-methylputrescine oxidase from tobacco. Plant & cell physiology 2007, 48(3):550-554.

60. Chintapakorn Y, Hamill JD: Antisense-mediated reduction in ADC activity causes minor alterations in the alkaloid profile of cultured hairy roots and regenerated transgenic plants of Nicotiana tabacum. Phytochemistry 2007, 68(19):2465-2479.

61. DeBoer KD, Dalton HL, Edward FJ, Hamill JD: RNAi-mediated down-regulation of ornithine decarboxylase (ODC) leads to reduced nicotine and increased anatabine levels in transgenic Nicotiana tabacum L. Phytochemistry 2011, 72(4-5):344-355.

62. Kato K, Shoji T, Hashimoto T: Tobacco nicotine uptake permease regulates the expression of a key transcription factor gene in the nicotine biosynthesis pathway. Plant physiology 2014, 166(4):2195-2204.

63. Hildreth SB, Gehman EA, Yang H, Lu RH, Ritesh KC, Harich KC, Yu S, Lin J, Sandoe JL, Okumoto S et al: Tobacco nicotine uptake permease (NUP1) affects alkaloid metabolism. Proceedings of the National Academy of Sciences of the United States of America 2011, 108(44):18179-18184.

64. Goossens A, Hakkinen ST, Laakso I, Seppanen-Laakso T, Biondi S, De Sutter V, Lammertyn F, Nuutila AM, Soderlund H, Zabeau M et al: A functional genomics approach toward the understanding of secondary metabolism in plant cells. Proceedings of the National Academy of Sciences of the United States of America 2003, 100(14):8595-8600.

65. Morita M, Shitan N, Sawada K, Van Montagu MC, Inze D, Rischer H, Goossens A, Oksman-Caldentey KM, Moriyama Y, Yazaki K: Vacuolar transport of nicotine is mediated by a multidrug and toxic compound extrusion (MATE) transporter in Nicotiana tabacum. Proceedings of the National Academy of Sciences of the United States of America 2009, 106(7):2447-2452.

66. Takahashi H, Kopriva S, Giordano M, Saito K, Hell R: Sulfur assimilation in photosynthetic organisms: molecular functions and regulations of transporters and assimilatory enzymes. Annual review of plant biology 2011, 62:157-184.

67. Azevedo RA, Arruda P, Turner WL, Lea PJ: The biosynthesis and metabolism of the aspartate derived amino acids in higher plants. Phytochemistry 1997, 46(3):395-419.

68. Shitan N, Hayashida M, Yazaki K: Translocation and accumulation of nicotine via distinct spatio-temporal regulation of nicotine transporters in Nicotiana tabacum. Plant signaling & behavior 2015, 10(7):e1035852.

**Table 1** Heterosis of nicotine content of hybrid combination（Va116×Basma）in tobacco

| Years | Developmental stage | Mid-parent heterosis | Over high-parent heterosis | Below low-parent heterosis |
| --- | --- | --- | --- | --- |
| 2013 | BT | 11.48 | 3.92 | 20.24 |
|  | AT | 34.56 | 24.65 | 46.18 |
| 2014 | BT | 10.39 | 2.13 | 20.11 |
|  | AT | 40.84 | 28.38 | 55.99 |
| 2015 | BT | 2.83 | 2.36 | 3.31 |
|  | AT | 44.82 | 33.48 | 58.26 |

Table 2 Comparison results of clean sequences on reference genomes

| Sample | Clean reads | Total mapped | Multiple mapped | Uniquely mapped | Reads map to + | Reads map to - | Non-splice reads | Splice reads |
| --- | --- | --- | --- | --- | --- | --- | --- | --- |
| VA116 | 69282386 | 56937342 (82.18%) | 1149403 (1.66%) | 55787939 (80.52%) | 27915758 (40.29%) | 27872181 (40.23%) | 39876131 (57.56%) | 15911808 (22.97%) |
| Basma | 76348022 | 63090417 (82.64%) | 1516187 (1.99%) | 61574230 (80.65%) | 30785508 (40.32%) | 30788722 (40.33%) | 40938897 (53.62%) | 20635333 (27.03%) |
| F1 | 66926392 | 53935066 (80.59%) | 1303116 (1.95%) | 52631950 (78.64%) | 26330586 (39.34%) | 26301364 (39.3%) | 34462915 (51.49%) | 18169035 (27.15%) |

(1) Total mapped represents the total amount of sequencing sequences that can be mapped to the genome. (2) Multiple mapped represents the total amount of sequencing sequences with multiple alignment positions on the reference sequence. (3) Uniquely mapped represents the total amount of sequencing sequences with unique alignment position on the reference sequence. (4) Reads map to '+', Reads map to '-' represent the number of sequencing sequence that aligne to positive and negative chains on the genome.

Table 3 The DEG counts of gene differential expression analyses

| Group | Up-regulated DEGs | Down-regulated DEGs | Up-regulated DEGs | Down-regulated DEGs |
| --- | --- | --- | --- | --- |
|  | (qvalue≤0.05) | | (qvalue≤0.05 and FC≥2) | |
| Basam vs. Va116 | 3255 | 3030 | 2951 | 2201 |
| F1 vs. Basam | 1231 | 986 | 797 | 791 |
| F1 vs. Va116 | 3888 | 2908 | 3292 | 2612 |

Table 4 KEGG pathway enrichment of genes showing dominance or transgressive regulation in hybrid

| Genetic hypotheses | Regulation pattern | KEGGID | Count | Size | Term | Padjust |
| --- | --- | --- | --- | --- | --- | --- |
| Dominance | Down | 970 | 15 | 24 | Aminoacyl-tRNA biosynthesis | 0.000828 |
|  | UP | 940 | 69 | 155 | Phenylpropanoid biosynthesis | 7.87E-07 |
|  |  | 909 | 16 | 27 | Sesquiterpenoid and triterpenoid biosynthesis | 0.004545 |
|  |  | 360 | 21 | 41 | Phenylalanine metabolism | 0.005072 |
| Overdominance | Down | 630 | 32 | 80 | Glyoxylate and dicarboxylate metabolism | 9.57E-16 |
|  |  | 30 | 15 | 64 | Pentose phosphate pathway | 0.000399 |
|  |  | 51 | 11 | 46 | Fructose and mannose metabolism | 0.003397 |
|  |  | 670 | 5 | 10 | One carbon pool by folate | 0.003452 |
|  |  | 730 | 4 | 7 | Thiamine metabolism | 0.00693 |
|  | UP | 960 | 16 | 29 | Tropane, piperidine and pyridine alkaloid biosynthesis | 4.65E-08 |
|  |  | 940 | 36 | 155 | Phenylpropanoid biosynthesis | 3.09E-06 |
|  |  | 1230 | 34 | 174 | Biosynthesis of amino acids | 0.00028 |
|  |  | 531 | 5 | 7 | Glycosaminoglycan degradation | 0.002295 |
|  |  | 760 | 7 | 15 | Nicotinate and nicotinamide metabolism | 0.002986 |
|  |  | 61 | 10 | 33 | Fatty acid biosynthesis | 0.007455 |
|  |  | 945 | 8 | 23 | Stilbenoid, diarylheptanoid and gingerol biosynthesis | 0.009065 |
|  |  | 450 | 6 | 16 | Selenocompound metabolism | 0.019903 |
|  |  | 460 | 9 | 33 | Cyanoamino acid metabolism | 0.019903 |
|  |  | 620 | 14 | 67 | Pyruvate metabolism | 0.020196 |
|  |  | 780 | 5 | 12 | Biotin metabolism | 0.022967 |
|  |  | 904 | 4 | 8 | Diterpenoid biosynthesis | 0.026009 |
|  |  | 270 | 14 | 72 | Cysteine and methionine metabolism | 0.029642 |
|  |  | 950 | 6 | 19 | Isoquinoline alkaloid biosynthesis | 0.033853 |
|  |  | 750 | 4 | 9 | Vitamin B6 metabolism | 0.036257 |
|  |  | 253 | 3 | 5 | Tetracycline biosynthesis | 0.038749 |
|  |  | 604 | 2 | 2 | Glycosphingolipid biosynthesis - ganglio series | 0.044164 |
