# Supplementary material for: Transcriptomic analysis reveals overdominance playing a critical role in nicotine heterosis in Nicotiana tabacum L
Source: BMC Plant Biol. 2018 Mar 22;18:48. doi: 10.1186/s12870-018-1257-x (PMC5863848; doi:10.1186/s12870-018-1257-x)
Supplement: Supplementary file 2 — Table S2. Quality assessment of sequencing data. (DOCX 13 kb) [file 12870_2018_1257_MOESM2_ESM.docx]

Table S1 Primer sequences of genes used in real-time PCR analysis

| Gene name | Encoded protein | Primer sequence | Reverse primer | Length（bp） |
| --- | --- | --- | --- | --- |
| gene_24753 | Actin-1 | GTGTTATGGTCGGAATGGG | TGGAACGCTAAATGTCTCAAA | 266 |
| gene_37484 | Actin-2 | CATGAAGATTAAAGGCGGAGTG | AACAGTTTGGTTGGAGTTCTGG | 199 |
| gene_45863 | ODC | ATACTAAAGCTGCTGGGTCC | AAAACCATCAAAACCACGGTAG | 128 |
| gene_60831 | ADC | GCGGAGACGCTCAACGAA | TGGTAAGTGCGGACAGGAT | 227 |
| gene_48826 | PMT | CCTACAATACGTCGCATTTCAC | CTTTCGCCAGAAGTATGATCG | 122 |
| gene_16500 | QPT | CCGACTTCATTCTCAGGC | GTTTCCCATCAAGCACAA | 158 |
| gene_42086 | MPO | TGCGAACTATCTGAACTTGA | ATACTGTCCTTAACTATCCTTCTT | 121 |
| gene_27718 | Cysteine synthase | CCCAAATACCTTCCTGGAAC | TAACAGCAATATAAGCACCACA | 115 |
| gene_28609 | AdoMet:Met S-methyltransferase | CATTCTGGCGTTTCAGTG | AGCATGGCTTCTCTGATATT | 122 |
| gene_74821 | S-adenosylmethionine synthas | ATGAGACTGTCACAAACGA | CGATGGGTTAAGGTGGAATATG | 113 |
| gene_21058 | Met synthase | GAGAACTCACGTTCCGAT | CTTGGAGAGTGGATGTCATAGA | 101 |

Table S2 Quality assessment of sequencing data

| Sample name | Raw reads | Clean reads | Clean  bases | Error rate(%) | Q20  (%) | Q30  (%) | GC content  (%) |
| --- | --- | --- | --- | --- | --- | --- | --- |
| Va116 | 72523116 | 69282386 | 10.39 | 0.02 | 96.64 | 92.03 | 42.02 |
| Basam | 79152084 | 76348022 | 11.45 | 0.02 | 96.67 | 92.07 | 43.66 |
| Va116×Basam | 69609816 | 66926392 | 10.04 | 0.02 | 96.25 | 91.28 | 44.29 |

Table S3 Number of genes with different expression levels.

| FPKM value | Va116 | Basam | Va116*Basam |
| --- | --- | --- | --- |
| 0~1 | 19792(36.72%) | 19655(36.47%) | 17868(33.15%) |
| 1~3 | 11676(21.67%) | 11491(21.32%) | 11852(21.99%) |
| 3~15 | 15179(28.17%) | 15756(29.24%) | 16655(30.90%) |
| 15~60 | 5364(9.95%) | 5245(9.73%) | 5662(10.51%) |
| >60 | 1882(3.49%) | 1746(3.24%) | 1856(3.44%) |
